# Remote sensing based forest cover classification using machine learning

**DOI:** 10.1038/s41598-023-50863-1

**Published:** 2024-01-02

**Authors:** Gouhar Aziz, Nasru Minallah, Aamir Saeed, Jaroslav Frnda, Waleed Khan

**Affiliations:** 1 Department of Computer Science and Information Technology, University of Engineering and Technology, Peshawar, Pakistan; 2 Department of Quantitative Methods and Economic Informatics, Faculty of Operation and Economics of Transport and Communication, University of Zilina, Zilina, Slovakia; 3 National Centre for Big Data and Cloud Computing, University of Engineering and Technology, Peshawar, Pakistan; 4 Department of Telecommunications, Faculty of Electrical Engineering and Computer Science, VSB Technical University of Ostrava, 70800 Ostrava, Czech Republic

**Keywords:** Environmental sciences, Engineering

## Abstract

Pakistan falls significantly below the recommended forest coverage level of 20 to 30 percent of total area, with less than 6 percent of its land under forest cover. This deficiency is primarily attributed to illicit deforestation for wood and charcoal, coupled with a failure to embrace advanced techniques for forest estimation, monitoring, and supervision. Remote sensing techniques leveraging Sentinel-2 satellite images were employed. Both single-layer stacked images and temporal layer stacked images from various dates were utilized for forest classification. The application of an artificial neural network (ANN) supervised classification algorithm yielded notable results. Using a single-layer stacked image from Sentinel-2, an impressive 91.37% training overall accuracy and 0.865 kappa coefficient were achieved, along with 93.77% testing overall accuracy and a 0.902 kappa coefficient. Furthermore, the temporal layer stacked image approach demonstrated even better results. This method yielded 98.07% overall training accuracy, 97.75% overall testing accuracy, and kappa coefficients of 0.970 and 0.965, respectively. The random forest (RF) algorithm, when applied, achieved 99.12% overall training accuracy, 92.90% testing accuracy, and kappa coefficients of 0.986 and 0.882. Notably, with the temporal layer stacked image of the Sentinel-2 satellite, the RF algorithm reached exceptional performance with 99.79% training accuracy, 96.98% validation accuracy, and kappa coefficients of 0.996 and 0.954. In terms of forest cover estimation, the ANN algorithm identified 31.07% total forest coverage in the District Abbottabad region. In comparison, the RF algorithm recorded a slightly higher 31.17% of the total forested area. This research highlights the potential of advanced remote sensing techniques and machine learning algorithms in improving forest cover assessment and monitoring strategies.

## Introduction

### Background

The forest ecosystem plays a vital role in preserving environmental equilibrium through pollution mitigation, flood regulation, and soil erosion prevention. The Food and Agricultural Organization recommends a forest cover of 20–30% for a country^1^. Pakistan has a limited forest cover, comprising 5.1 percent of the total land area, equivalent to 4.478 million hectares^2^. This translates to just 0.021 hectares per person, significantly below the global average of 1 hectare per person. Over the past thirty years, over 60 percent of the Himalayan Forest has undergone destruction^3^. The scarcity of forests in Pakistan can be attributed to the rapid growth of population and poverty, coupled with a lack of awareness among the people. The primary drivers of deforestation in the country are the extraction of wood, fuel, and charcoal by the local population^2^. However, Certain regions in Pakistan, including Mansehra, Abbottabad and Swat, boast rich biodiversity with over 430 tree species. The conventional and manual approaches to supervising forests present challenges in terms of being time-consuming, expensive, and labour-intensive. The task of physically visiting forests to document information about each tree is both challenging and costly. Monitoring becomes particularly challenging in hilly areas, especially during harsh and cold weather conditions when these areas are covered in snow. In this age of technological progress, it is essential for the government to prioritize the integration of advanced and scientific technologies, such as Remote Sensing^4^, to effectively manage deforestation.

### Remote sensing technologies

Remote Sensing employs satellites and sensors to study the Earth's surface, providing valuable information from a distance. Commercial satellites like Sentinel-2, Modis, and Landsat offer enhanced spatial, spectral, and temporal resolution, providing open data for remote sensing. Sentinel-2, with its 13 multispectral bands, including the vegetation red edge bands, offers 5 days of temporal data for regular analysis. Sentinel-2's capabilities make it well-suited for detailed forest analysis, change detection, and comprehensive feature analysis. Various techniques simplify the estimation process in remote sensing, yielding notably accurate results in detecting and estimating different forest types, offering insights into their health and maturity. In our research, we utilized Sentinel-2's temporal data for forest analysis, benefiting from its features as an open data satellite. Sentinel-2 proves to be a valuable resource, contributing to an enhanced understanding of forests, encompassing their health and maturity status.

Our designated study area encompasses District Abbottabad, located within the Hazara Division of the Khyber Pakhtunkhwa province in Pakistan. This district falls under the Wet Mountains Agri Ecozone, featuring verdant hills and is widely recognized as a popular summer resort destination. Through the utilization of temporal data from Sentinel-2, our research yielded noteworthy results. Equation (1) provides the formula for calculating the area of the Sentinel-2 image.

1 $$Area= \frac{(Total Pixel\times100 {m}^{2})}{1000\times1000}\times100=Hectares$$

Li et al. employed multispectral Sentinel-2 satellite imagery^5^ to evaluate the effectiveness of forest-type mapping in Shangri-La, the administrative region of Yunnan Province, China. They applied the Random Forest algorithm within the Google Earth Engine (GEE)^5^, with a primary focus on identifying and detecting various forest types. The study aimed to assess the Random Forest algorithm's efficacy within the GEE platform and distinguish variations in the main forest types across an extensive area. Furthermore, the research aimed to estimate critical features for forest classification. The analysis successfully identified eight distinct forest cover types, achieving a 95.76% accuracy in distinguishing between forest and non-forest areas, along with a Kappa coefficient of 91.34%. The utilization of the Google Earth Engine platform played a pivotal role in effectively monitoring the dynamic changes in forest cover.

Conventional approaches for monitoring, classifying, and estimating tobacco crop yield are expensive and time-consuming. The absence of an advanced system utilizing state-of-the-art remote sensing technologies for monitoring, classification, and yield estimation of tobacco crops was evident in Pakistan. To bridge this gap, Khan et al. in collaboration^4^ with the Pakistan Tobacco Board (PTB), conducted research to establish an innovative machine learning mechanism. They employed temporally layer-stacked Sentinel-2 satellite data to estimate tobacco crops in Pakistan. For the detection of tobacco crops, the researchers devised a model based on an Artificial Neural Network. Implementing the Artificial Neural Network classifier with a single image^4^ achieved an Overall accuracy of 88.49%, which was further improved to 90.45% through the application of NDVI stacking. Notably, through experiments with temporally stacked imagery, they attained an Overall accuracy of 95.81%, marking a significant 7.32% improvement over the benchmark scheme.

Like many other countries, China experiences the effects of Land Use Land Cover (LULC) changes. In tackling this challenge in the Ganan Prefecture from 2000 to 2018, Liu et al.^6^ utilized the dense time stacking of multi-temporal Landsat images and implemented the random forest algorithm on the Google Earth Engine (GEE) platform for LULC mapping. The classification accuracy for the entire dataset fell within the range of 89.14% to 91.41% and Kappa Coefficient 0.86. The primary land use and land cover (LULC) categories in the study area were grassland, making up 50% of the total area, and forest, encompassing 25%.

Forest dynamics result from various factors, with seasonal influences playing a significant role. In response, Jiang et al.^7^ introduced Forest-CD, a model that utilizes high-resolution images (VHR). This model employs an encoder–decoder architecture, integrating background information. The encoder, driven by the Swin Transformer, systematically extracts change features, effectively mimicking global information. Conversely, the Forest Change Detection decoder employs the feature pyramid network to recover fused information and feature scales at different levels. Analysis of an extensive forest dataset indicates that the Forest-CD network, utilizing VHR images, attains a higher F1 score. Additionally, the outcomes from Forest-CD demonstrate a decrease in pseudo changes.

The Random Forest machine learning algorithms find frequent applications in data classification^8,9^, object recognition^10,11^, and image segmentation^12^. Feng et al.^13^ introduced a novel training sample selection method specifically designed for Random Forest Modeling in greenhouse identification using Super View-1 imagery. This innovative approach enhances classification accuracy and generalization capabilities. The new Random Forest Modeling allows for the automatic selection of high-quality training samples, resulting in high-precision classification. Furthermore, the researchers anticipate that this improved and advanced Random Forest model can extend its utility to identify various ground objects such as roads and buildings.

To address challenges in advancing Remote Sensing technologies, Benson et al.^14^ introduced multimodal remote sensing model for forest parameter estimation. This approach utilizes Light Detection and Ranging (LiDAR), polarimetric radar, and near-infrared passive optical sensing platforms, coupled with physics-based models. These models prove beneficial in precisely estimating aboveground biomass and measuring Canopy Height in homogeneous areas. The forest parameter estimation algorithm employs a combination of geometric and electromagnetic sensor model methods. Despite having minimal input information, this integrated method yields accurate results for estimating forest structure, along with minimal root mean square errors.

In order to precisely map and identify spatiotemporal changes Erfanifard et al.^15^ carried out a three-decade study in Iran with a focus on mangrove habitats. The Submerged Mangrove Recognition Index (SMRI), a recently developed technique, and Landsat data from 1990 to 2020 were used in the study. In the process of Mangrove mapping, the researchers utilized four vegetation indices in conjunction with eight mangrove-specific indices. The study found SMRI to be a particularly effective index. Utilizing long-term Landsat data, the estimated mangrove coverage in Iran was approximately 13,000 ha in the year 2020.

Wallner et al.^16^ addressed the dynamic and impactful changes occurring in the Central European forest ecosystem, driven by climate uncertainties and shifts in weather patterns. In response to this, they employed satellite data from ZiYuan-3 (ZY-3) within a Remote Sensing-guided Forest inventory framework. The objective was to reduce the required field sample size while analysing the standard grid inventory. The utilization of 3D ZY-3 demonstrated its suitability in supporting forest inventory by effectively minimizing sample size and enhancing inventory frequencies.

Sundarban a mangrove forest situated at Nijhum National Park^17^ faces challenges in the degradation of the forest cover. A study was conducted by Islam et al. in NDP to find out the decades' changes in the mangrove forest by using GIS tools and remote sensing available data. They used maximum likelihood classification techniques by using Landsat images of 3 decades from 1990 to 2020. SAVI and NDVI-based classification is performed for forest cover changes in comparison with supervised classification. With this work, they find out that in the first decade from 1990 to 2000 almost one-third of deforestation occurred. However, in the last decade increase of 310.32 ha have recorded in mangrove forest cover.

Deforestation changes the forest structure, functionality, and ecosystem process^18^. Challenges facing deforestation are the estimation of emissions and identifying the area affected and the total amount of biomass lost. Till now no reliable method is established to identify the causes of deforestation to monitor forest fire, cattle grazing, and fuelwood collection. High spatial and temporal images are used to detect small-scale disturbances. However, using high-resolution images is costly too. For forest fires and detecting logging, Gao et al.^18^ suggested the SMA Remote sensing method for the detection of deforestation. To measure the intensity of deforestation Lidar and radar are suitable because of their capacity to measure the 3D of the forest structure and biomass measurement.

In the last decades, deforestation and woodland is greatly affected by natural disasters^19^. To detect the early smoke and flame various remote sensing technologies systems and algorithms are used by Barmpoutis et al.^19^. Terrestrial, airborne, and spaceborne-based systems are identified. Large Earth Observation Satellite proved to be successful in wide-range broadcasting in early smoke and flame detection. CubeSats is a low-Earth-Orbiting satellite that has a significant advantage over traditional satellites in smoke detection and fire detection, they are also economical, better in temporal resolution, have good response time, and in better coverage.

A Spatio-temporal study has been conducted by Negassa et al.^20^ on Kotmo forest which is situated in the Guto Gita District of the East Wollega zone of Ethiopia to find the status of the Forest cover by using the GIS and Remote Sensing techniques. By using geospatial techniques, it is recorded that the total area of dense forest in Kotmo forest was 32.73% in 1991. Which decreased to 26.16% in 2002. The forest further decreased to 20.5% in 2019. A decrease in the open forest is also recorded i.e., 18.19% and 16.14% in 1991 and 2019 simultaneously. However, a considerable amount of increase in agricultural land is recorded from 24.78% in 1991 to 29.21% and 33.50% in the years 2002 and 2019, respectively. This study suggests policy interventions to protect the Kotmo forest priority area from loss and degradation.

Biomass mapping is a vital and practical tool in the realm of forest management, particularly for monitoring forests and evaluating deforestation processes. For this purpose, Sharifi et al.^21^ conducted a study aimed to employ Multivariate Relevance Vector Regression (MVRVR) as a Bayesian model with a kernel-based framework for predicting above-ground biomass (AGB) in the Hyrcanian forests of Iran. Using field data and multi-temporal PALSAR backscatter values for Training and Testing, the researchers compared the results with alternative methods such as multivariate linear regression (MLR), multilayer perceptron neural network (MLPNN), and support vector regression (SVR). The findings revealed that the SVR model outperformed others, especially at the lowest saturation point. The MVRVR model significantly enhanced AGB estimation precision, showing exceptional performance, particularly in situations involving the maximum saturation point.

In the realm of remote sensing, hyperspectral images (HSIs) distinguish themselves as a valuable source of information, owing to their distinctive features applicable in various contexts. But still due to many reasons the hyperspectral images performance are reducing due to many reasons especially to the limited number of samples. In order to improve the HIS accuracy Ghaderizadeh et al.^22^ proposed the creation of a classification model for hyperspectral images (HSI) named MDBRSSN, an acronym for Multiscale Dual-Branch Residual Spectral–Spatial Network with Attention. The proposed model underwent experiments on four datasets, showcasing its excellence compared to state-of-the-art methods, particularly in scenarios with a restricted number of Training samples. The proposed model achieved Overall accuracies of 99.64%, 98.93%, 98.17%, and 96.57% with only 1%, 1%, 5%, and 5% of labelled data for Training, respectively. These results surpass those of state-of-the-art methods.

Various factors, including flooding, contribute to deforestation. The importance of implementing a real-time monitoring system for evaluating flood risks and improving disaster response times cannot be overstated. In a study conducted by A. Shirifi^23^, the classification of SAR data involved employing thresholding, machine learning algorithms, and an object-based method. The thresholding process played a crucial role in identifying flooded regions. Upon comparing the results, the machine learning algorithm exhibited significant success. These findings highlight the importance of Sentinel-1 images as crucial data for refining methodological guides, indicating their potential as a novel resource for monitoring flood risks.

Remote sensing proves beneficial in flood control efforts, as floods can contribute to deforestation. Floods pose a potential threat in numerous locations, with heightened susceptibility observed in forests, the agricultural industry, and infrastructure situated near rivers. This vulnerability is attributed to the widespread impact of floods on forests and agricultural land across diverse areas. Tariq et al.^24^ implemented an experimental approach to evaluate the vulnerability of flood mapping in the northern areas of Punjab, Pakistan, through the integration of FR and AHP techniques. Eight parameters were deliberately selected to determine the weight of relative significance, employing pairwise matrix correlation. Six parameters are from Remote Sensing imagery including Sentinel-2 satellite. The flood hazard map was generated using ArcGIS algorithms to identify the extremely high, moderate, and low flood zones in the final output.

Several research studies have recommended the utilization of remote sensing imagery for environmental monitoring. For this purpose Mohammadi et al.^25^ employed Sentinel-1 SAR data, along with the utilization of Sentinel-2 imagery, to promptly identify oil spills in the Persian Gulf. They employed VV-polarized images from Sentinel-1 SAR data to illustrate the existence of oil patches. Sentinel-2 data distinguishes itself as a highly effective sensor for detecting oil slicks, thanks to its exceptional spatial, spectral, and temporal resolution. They recommended that If users lack access to field data, it is advisable to employ the OBIA method to assess the accuracy of results derived from SAR data.

Zaman et al.^26^ conducted a study with the goal of determining the ideal zones for saffron cultivation in Miyaneh. The research utilized Landsat 8 satellite images and applied the Weighted Linear Combination (WLC) method. The study period extended from November 2019 to May 2020. The results indicated that the prime locations for saffron cultivation in the examined area are concentrated in a strip running from the southwest to the southeast, along with specific northern regions.

Sharifi et al.^27^ in the field of remote sensing advocated for the use of Polarimetric Synthetic Aperture Radar technology (PolSAR) when SAR images face challenges due to speckle noise. They highlighted the effectiveness of PolSAR in capturing images across different polarizations as a practical and alternative solution. The Fast ICA method is strongly endorsed for its proficiency in reducing speckle, preserving details, and demonstrating remarkable speed.

Kossari et al.^28^ introduced a rapid method for dimensioning the Attitude Determination and Control System (ADCS) of Earth observation satellites. They applied a matching diagram technique, well-established in aircraft industries for aircraft design. The study emphasized spatial and temporal resolutions as the key performance requirements (PRs).

Yuh et al.^29^ conducted a comparative analysis of four distinct machine learning algorithms to monitor changes in Land Use and Land Cover (LULC) in northern Cameroon. Their study utilized Landsat 7 ETM and Landsat 8 OLI imagery from November 2000 and November 2020. KNN, SVM, RF, and ANN were among the algorithms that were assessed, all of them showed a commendable level of accuracy. The KNN algorithm produced a Kappa Coefficient of 89% and an Overall Accuracy of 91.1% for the year 2020. Likewise, the ANN algorithm produced a high 94% Kappa Coefficient along with a high 95.8% Overall Accuracy. The RF algorithm demonstrated a 94% Kappa Coefficient and an Overall Accuracy of 90.3%. With a Kappa Coefficient of 87%, the Overall accuracy for SVM was 88.6%. The study's conclusions showed that there was a notable reduction in the amount of forest cover between 2000 and 2020 as a result of the conversion of these forested regions into agricultural land, mostly for the production of crops.

Moradi et al.^30^ explored changes in forest cover in the Zagros Mountains, Western Iran, utilizing Landsat imagery. They applied a CNN deep learning algorithm to discern alterations in the landscape. The results of their study revealed a substantial decline in forest cover over the past thirty years. The CNN algorithm proved effective in distinguishing oak forest from water and agricultural classes. Their research achieved a high accuracy of 97% and a Kappa coefficient of 94.7% when utilizing Landsat TM imagery. Similarly, with Landsat ETM imagery, they attained a 95% Overall accuracy and a Kappa coefficient of 94.1%.

This work is organized as follows. The methods and material are discussed in “Methods and material” while the results of our experimentation are discussed in “Experiments and results”. Discussion on our proposed algorithms and obtained results are entailed in “Discussion”. Lastly, we succinctly conclude in “Conclusion” along with some future propositions.

## Methods and material

To initiate the forest cover detection project, the Abbottabad region has been designated as the pilot area. This geographically diverse area is characterized by rolling hills and enveloped by lush green mountains, making it a renowned summer retreat admired for its forested charm. The process of gathering accurate data and geographical points unfolded in multiple stages. Initially, on-site inspections are conducted to categorize the classes, and the following four classes are chosen.

i.Fields

ii.Forest

iii.Urban area

iv.Shrubs.

**Figure 1:**
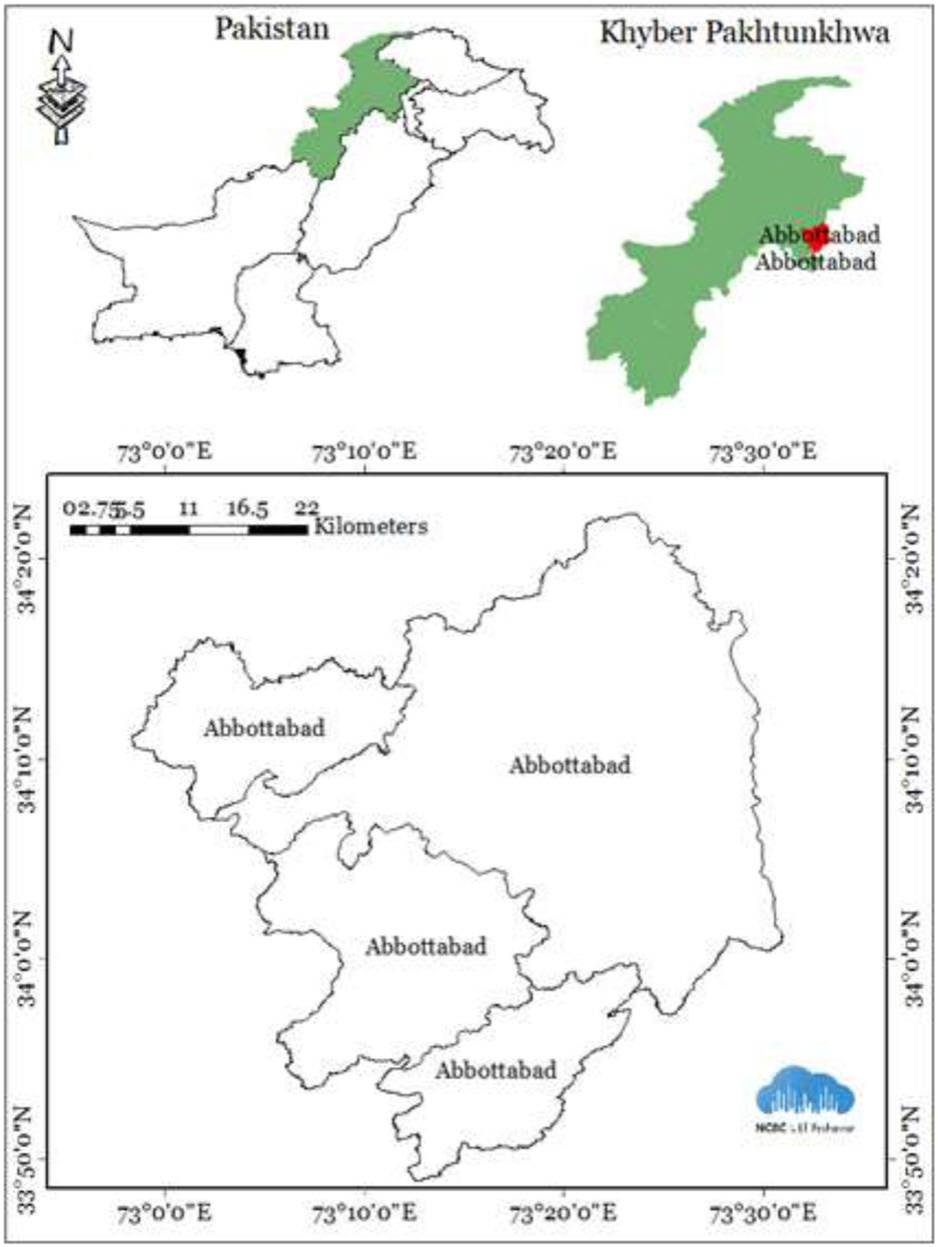
District Abbottabad shape file.

Secondly, to enhance the reliability of the data, the shape file for Abbottabad is acquired from the Pakistan Forest Institute in Peshawar, a well-respected organization in the country. To ensure data accuracy and utilize cutting-edge technology, the "Geosurvey App," an indigenous application developed by the National Center of Big Data and Cloud Computing (NCBC) in Peshawar (https://www.ncbcpeshawar.com), is employed. Utilizing the “GeoSurvey App” for the Fields class, we meticulously choose and outline a total of 900 polygons. The Forest class comprises 901 carefully selected polygons. Likewise, for the Urban class, we identify and pick 900 polygons. The data pertaining to Shrubs polygons is retrieved from the Forestry Planning and Monitoring System in Peshawar. Figure 1 displays the shape file for the Abbottabad district.

In the third phase, Sentinel-2 satellite images are acquired. The experimentation involves working with both a single downloaded image and a temporally sequenced downloaded image. Specifically, a Sentinel-2 single image from October 27th, 2021, for District Abbottabad is obtained. For the temporal image set, four images from September 2nd, 2021, October 27th, 2021, November 11th, 2021, and December 11th, 2021, are downloaded.

The following procedures are executed on these downloaded images:

1. Preprocessing is carried out using SNAP Desktop, with resampling parameters being configured.
2. The resampled data is subsequently employed for further processing.
3. All the images are layer-stacked.
4. A mask is constructed, and this mask is applied to extract the Abbottabad image from the shapefile.
5. A CSV file is generated for the region of interest (ROI).

**Figure 2:**
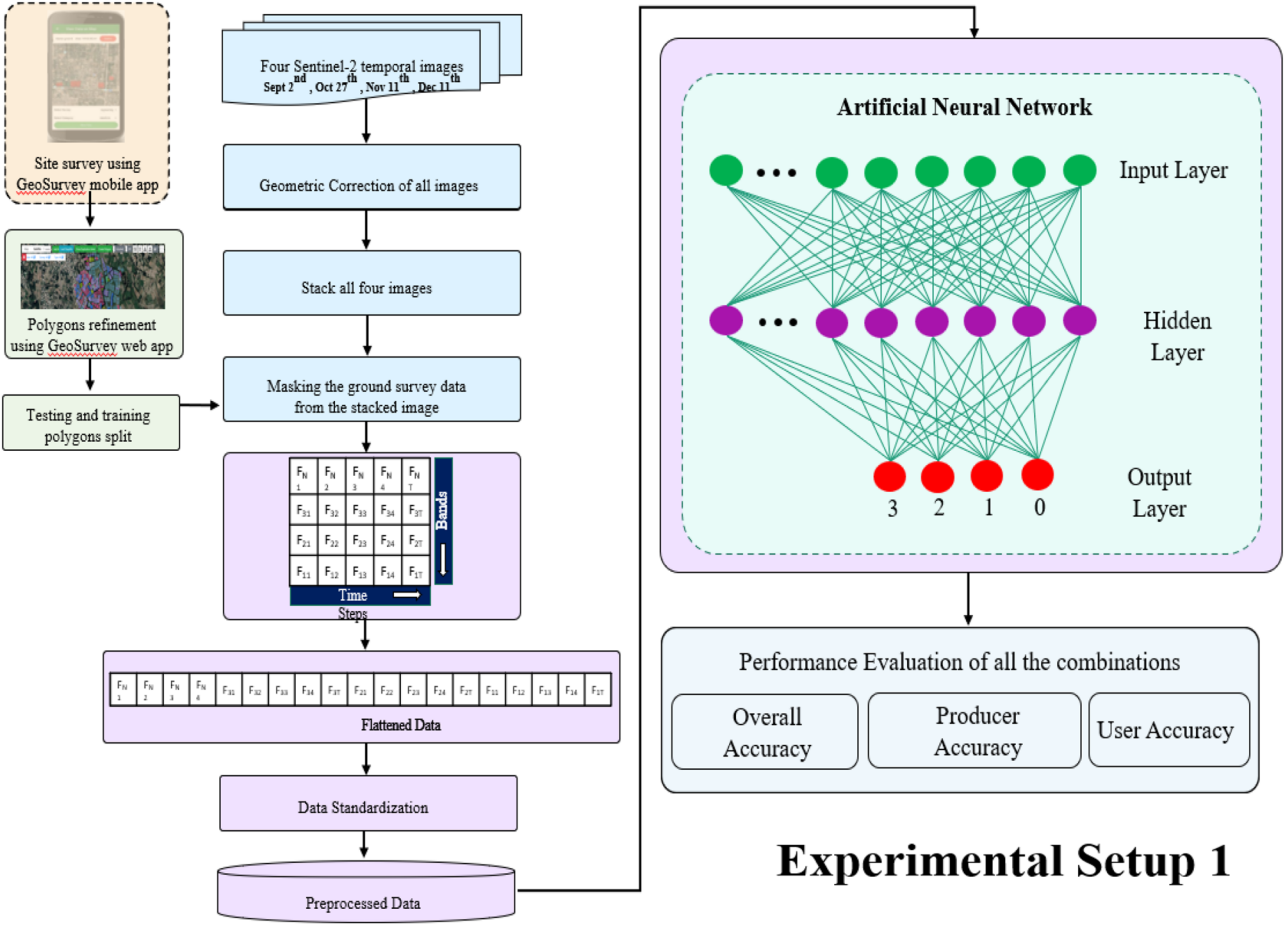
Artificial neural network algorithm methodology.

In the fourth phase, following the establishment of the Remote Sensing Dataset, experiments are conducted using Artificial Intelligence Neural Network algorithms with diverse parameters and Random Forest algorithm. Artificial Neural Networks (ANNs) proficiently manage diverse remote sensing data, incorporating both multispectral and hyperspectral imagery. Their versatility allows for seamless adaptation to the diverse spectral bands and resolutions commonly encountered in various remote sensing applications. Artificial Neural Networks (ANNs) have proven effective across diverse applications in remote sensing, such as land cover classification^8^, object detection^11^, vegetation and crops monitoring^4^, and terrain analysis^8^. Artificial Neural Networks find applications in various domains such as image processing and character recognition^31^, classification^32^, forecasting, enhancement^33^, analysis^34^, estimation, and prediction^35^. Their adaptability renders them suitable for a broad spectrum of tasks within the field. Specifically, networks with a significant number of parameters may be prone to overfitting, capturing noise or specific patterns in the Training data that may not generalize effectively to new, unseen data. The whole procedure is depicted in Fig. 2.

**Figure 3:**
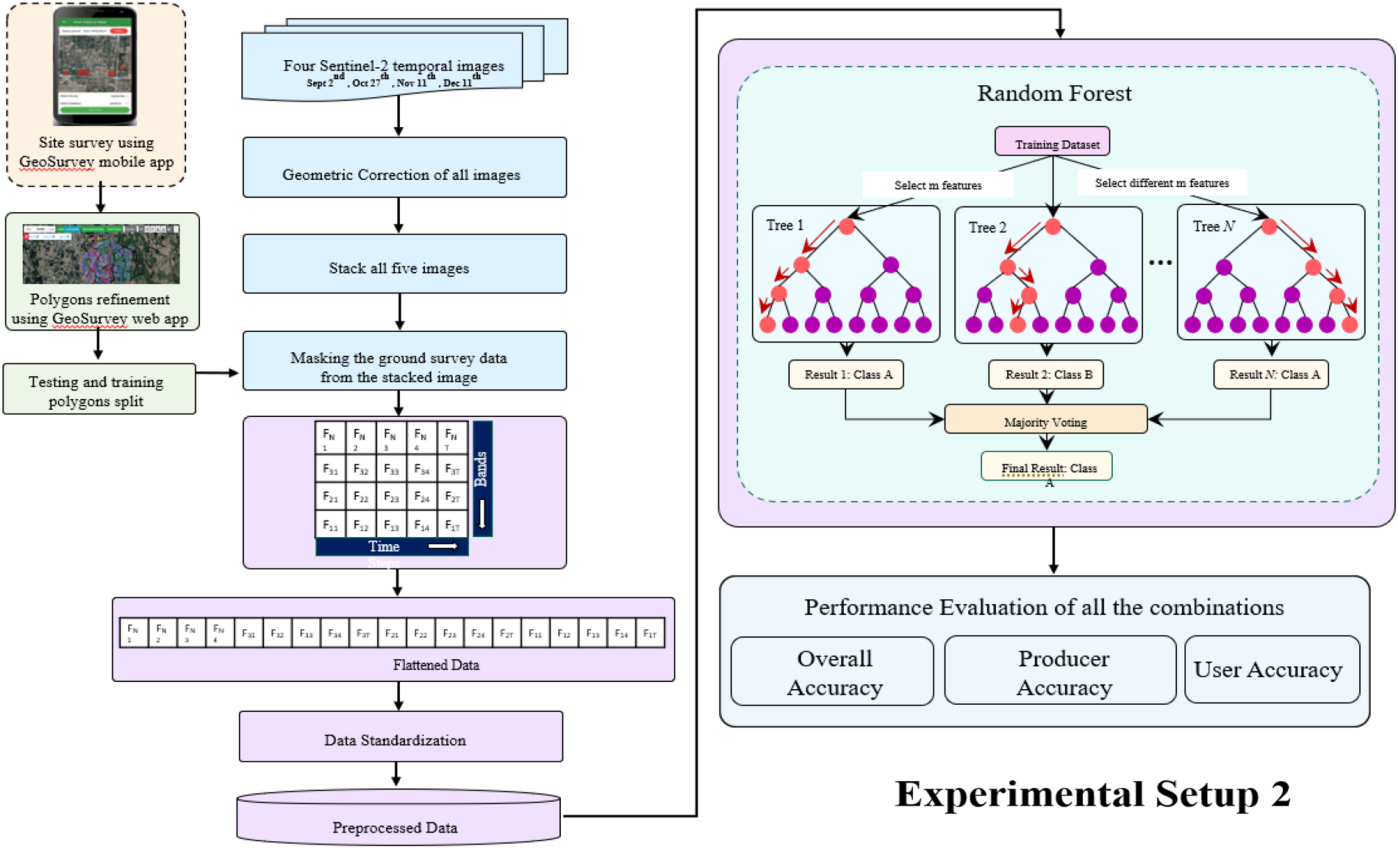
Artificial neural network algorithm methodology.

Within the machine learning domain, Random Forest (RF) is widely acknowledged as a frequently employed ensemble learning technique suitable for both classification and regression tasks. In this work, we opted for RF due to its basic ensembled structure and computational feasibility as compared to other bagging and boosting based ensemble learning techniques (i.e., XGBoost, CATBoost etc.). As it can be seen in Fig. 3, it operates as an ensemble model, generating multiple decision trees using randomly selected subsets of Training samples and variables. The Random Forest (RF) classifier demonstrates reduced sensitivity^36^ in comparison to other streamlined machine learning classifiers concerning the quality of Training samples and overfitting concerns. Random Forests may require substantial computational resources, especially when dealing with a substantial number of trees and features. The Training and evaluation of a large ensemble can be computationally demanding.

### Artificial neural networks

#### Classification

**Figure 4:**
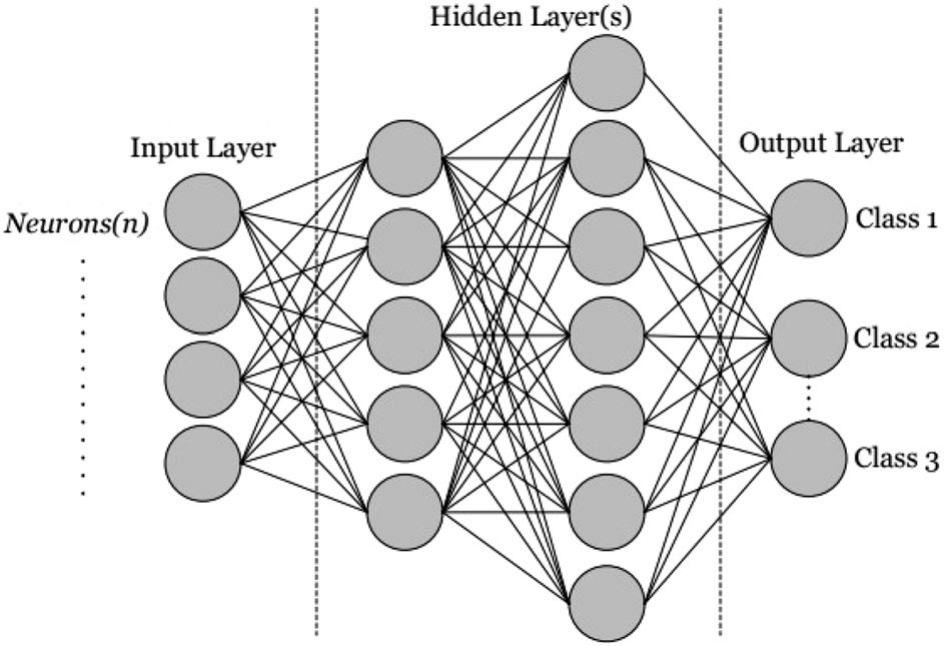
Artificial neural network algorithm.

Khan and Minallah^4^ employed the Artificial Neural Network algorithm in their study. They emphasized that artificial neurons serve as the fundamental components of Artificial Neural Networks^37^. For the implementation of a neural network, a minimum of three layers is required, namely the Input Layer, the Hidden Layer, and the Output Layer, as depicted in Fig. 4. The Input Layer transmits input to the Hidden Layer, also known as the middle layer, which addresses problems by utilizing multiple Processing Elements (PE). The Output Layer, the final layer, generates output based on given input parameters.

Initially, every Artificial Neural Network goes through Training to understand and compare its reactions when given new pixels, figuring out which side of a linear separating line they fall on^35^. After that, the processing part depends on the inputs and weights from the layer before. The Processing Element (PE) handles a set of inputs, like X =  × 1, × 2, × 3……x_N_, where w is the connection weight, θ is a bias, and Z0 is the Output Layer.

#### Feed forward neural network

A Feed-Forward Neural Network (FFNN) utilizes a layer of interconnected neurons for the processing and transmission of information. It falls under the category of Artificial Neural Networks that use a supervised classification method to approximate a classifier. During FFNN Training, adjustments are made to the weights at the nodes with the goal of reducing the disparity between the activation of the output nodes and the input. The network must learn the appropriate weights and biases to precisely classify the input data. Certain features of Feed-Forward Networks encompass:

- Processing Elements (PEs) are structured in layers, wherein the input layer accepts input data, the output layer produces outputs, and the intermediary layers, known as hidden layers, do not have external connections but exclusively interact with other layers within the model.
- Information travels in a single direction, moving from the input layer through the hidden layer and reaching the output layer.
- FFNNs are non-cyclic, signifying the absence of feedback connections in the network, which inhibits neurons from exchanging information with each other in a reverse manner.

Connections are established, with a Processing Element (PE) such as H_1_ connected to inputs x_1_, x_2_, and x_3_, and H_2_ linked to inputs x_1_, x_2_, and x_3_, as depicted in Figure 4. Equation (2) accounts for all the weights in play. PE computes the matrix product of the hidden layer with these weights, includes its own bias, and subsequently applies the activation function. The matrix computation is presented as***:***

$$=\left|\begin{array}{ccc}{W}_{11}& {W}_{12}& {W}_{13}\\ {W}_{21}& {W}_{22}& {W}_{23}\end{array}\right|* \left|\begin{array}{c}{x}_{1}\\ {x}_{2}\\ {x}_{3}\end{array}\right|$$

2 $$=\left|\begin{array}{l}{W}_{11}*{x}_{1}+{W}_{12}*{x}_{2}+{W}_{13}*{x}_{3}\\ {W}_{21}*{x}_{1}+{W}_{22}*{x}_{2}+{W}_{23}*{x}_{3}\end{array}\right|$$

3 $$ {\text{H}}_{{1}} = \, \left( {{\text{ W}}_{{{\text{IJ}}}} *{\text{ l}}_{{\text{i}}} + {\text{ B}}_{{\text{i}}} } \right) $$

4 $$O=({W}_{IJ}*H+B)$$

The Hidden layers' value is computed by adding up the products of the input values and their corresponding weights, as described in Eq. (3). The key purpose of this computation is to ascertain how the system should be adjusted to match the output with the desired target. Even slight modifications in weights can result in substantial changes in output^30^. This attribute facilitates the learning process.

#### Parameters for neural network

Before establishing the parameters, certain decisions must be made, including determining the number of layers to be employed. Generally, three layers are deemed satisfactory, with the first designated as the Input Layer, the subsequent one as the Hidden layer, and the last one as the Output layer. The input layer typically receives nodes corresponding to the number of components (features) in the pixel vectors. The following parameters in Table 1 have been set for the ANN algorithm.

**Table 1:** Parameters for ANN algorithm.

S. no.	Parameters	Value/type
1	Learning rate	0.01
2	Training momentum	0.900
3	Training RMS exit criteria	0.100
4	Number of hidden layer	2
5	Number of training iterations/epochs	50, 100, 200, 300
6	Activation function	Relu
7	No of neurons in hidden layer	64
8	Batch size	32

### Random forest algorithm

**Figure 5:**
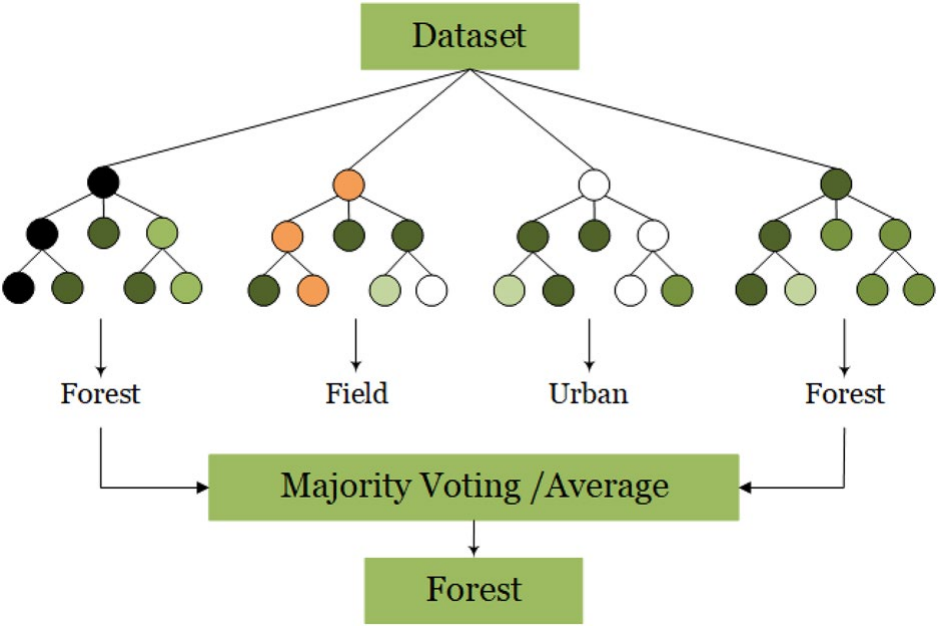
Random forest algorithm.

The Random Forest is a supervised classification machine learning algorithm that constructs and grows multiple decision trees to form a "forest." It is employed for both classification and regression problems shown in Fig. 5. In classification, it builds decision trees on various samples and takes a majority vote, while in regression, it calculates the average for different samples. A notable feature of the Random Forest Algorithm is its ability to handle datasets with categorical variables for classification, leading to improved results.

#### How random forest algorithm work

The Random Forest algorithm employs Bagging or Bootstrap Aggregation Techniques. Bagging entails generating multiple Training subsets from the sample Training data with replacement, and the ultimate output is decided by the majority of votes. Bootstrap randomly selects rows and features from the dataset to create sample datasets for each model. Aggregation consolidates these sample datasets through majority voting to generate the final output. Bootstrap Aggregation is effective in mitigating the variance of high-variance algorithms, like decision trees.

#### Steps involved in random forest algorithm

1. Random Forests operate on a given dataset with N records and K outputs, where N represents the number of samples, and K denotes the number of classes.
2. A decision tree is created for each set of samples to produce the output.
3. In classification, the final output is determined by assigning greater importance to the majority of votes.

Safira Desdhanty and Rustam^38^ implemented the Random Forest algorithm in their research. In their methodology, they define S = {(xi, yi)}, where xi represents the numerical feature, and yi corresponds to the respective labels. Assuming the Random Forest has T features, and P denotes the number of trees in the forest, N trees are randomly selected in the Random Forest, and each is employed to construct a decision tree. This process is repeated P times, and at each node, a small subset of features is created. The best feature for each subset is then determined. The outcome of this procedure is a selected feature A that achieves the highest score^38^: the algorithm is presented in the table below^38^.

**Table:** Algorithm Random forest.

Initialization: A training set *S*: = {(xi,yi)}, *T* features, and number of trees in forest *P* 1. Select *M* trees from the dataset, in order to to construct a decision tree 2. Redo the previous step* P* times 3. At each node: 4. Construct a small subset of *F*, call it *f* 5. Separate the most appropriate features in *f* 6. The category that gains the majority votes will be given a new record The Output will be the selected features that have the highest accuracy score	

The following Parameters have been set in Table 2 for the Random Forest algorithm.

**Table 2:** Parameter for random forest algorithm.

S. no.	Parameter	Value
1	Maximum depth	5, 10, 20

These outcomes play a crucial role in determining the Overall Accuracy and Kappa Coefficient for both the Training and Testing Data sets.

Overall accuracy stands out as a frequently used evaluation metric. It signifies the ratio of accurately classified instances, or data points, to the total number of instances in a dataset. This metric serves as a fundamental benchmark for assessing the model's performance in terms of correct classifications across the entire dataset. The formula for the Kappa coefficient is as follows in Eq. (5):

5 $$Overall\, Accuracy= \frac{Sum\, of\, Correctly\, Classified \,Pixels}{Total\, Number\, of\, Pixels}\times100\%$$

The Kappa coefficient, also known as Cohen's Kappa, is a statistic that measures the agreement between observed and expected classification results while considering the possibility of agreement occurring by chance. The formula for the Kappa coefficient is as follows in Eq. (6):

6 $$Kappa\, Coefficient=\frac{Overall\, Agrement-Chance \,Agreement}{1\,- \,Chance\, Agreement}$$

Overall Agreement is the proportion of observed agreement between the classified results and the reference (ground truth) data.

Chance Agreement is the expected agreement due to chance. It is calculated based on the marginal probabilities of agreement for each class.

Concerning the Training Data, as delineated in Table 3, we have selected 17,101 pixels for the Fields class, 33,045 pixels for the Forest class, 3678 pixels for the Shrubs class, and 9542 pixels for the Urban class. Regarding the Testing Data, as indicated in Table 1, 7377 pixels are chosen for the Fields class, 14,166 pixels for the Forest class, 2058 pixels for the Shrubs class, and 4191 pixels for the Urban class.

**Table 3:** Total number of training and testing pixels.

Class	Training pixel	Testing pixels
Fields	17,101	7377
Forest	33,045	14,166
Shrubs	3678	2058
Urban	9542	4191

Through the utilization of the Random Forest Supervised machine learning Classification Algorithm, we attained a Training Overall accuracy of 99.79% and Testing Overall accuracy of 97%. Furthermore, the application of the Artificial Neural Network Deep Learning algorithm resulted in remarkable outcomes, with a Training Overall accuracy of 98.06% and Testing Overall accuracy of 97.75%.

In the last step, the total forest-covered area is estimated. The detailed procedural steps for both the Artificial Neural Network (ANN) and Random Forest algorithms are illustrated in Figs. 4 and 5.

## Experiments and results

To perform forest cover classification based on remote sensing through machine learning and deep learning algorithms, experiments were carried out utilizing both the Neural Network classification algorithm and the Random Forest Classification algorithm.

Obtaining results from the stacked Sentinel-2 single-layer image using a Neural Network classification algorithm with varying numbers of Training iterations yielded exceptional outcomes, as depicted in Table 4. To ensure precise Overall accuracy and Kappa Coefficient, we allocated 70% of the data for Training in our experiments and reserved 30% for Testing or Validation purposes. The experimental findings demonstrate that with a total of 50 iterations, the Overall Training accuracy reaches 91.23%, and the Validation accuracy is 92.83%. The Kappa Coefficient for 50 iterations is 0.864 in the Training data and 0.889 in the Testing data. As the total number of iterations increases, a noticeable enhancement in the results is observed. With 100 iterations, the Training Overall accuracy is 90.94%, and the Validation accuracy is 92.54%. The Kappa Coefficient for 100 iterations is 0.854 in the Training data and 0.884 in the Testing data. Similarly, with 200 iterations, the Training Overall accuracy reaches 91.37%, and the Validation Overall accuracy is 93.77%. The Training Kappa Coefficient is 0.865, and the testing Kappa Coefficient is 0.902 for 200 iterations. Further increasing the total iterations to 300 yields a Training Overall accuracy of 91.91% and a testing Overall accuracy of 93.48%. The Kappa Coefficient for 300 Training iterations is 0.875, and for Testing 300 iterations, it is 0.899.

**Table 4:** ANN algorithm results for single layer stacked image.

ANN results for single layer stacked image											
Training 70							Testing 30				
No of training		Fields_ training	Forest_ training	Shrubs_ training	Urban_ training	Overall accuracy	Fields_ testing	Forest_ testing	Shrubs_ testing	Urban_ testing	Overall accuracy
50	Overall accuracy = 91.23%, kappa coefficient = 0.864						Overall accuracy = 92.83%, kappa coefficient = 0.889				
	Unclassified	0	0	0	0		0	0	0	0	
	Fields	15,922	791	1019	304	93.11%	6891	292	137	189	93.41%
	Forest	207	31,004	1149	17	93.83%	106	13,280	290	12	93.88%
	Shrubs	284	1135	3337	10	60.62%	111	550	1631	10	79.25%
	Urban	688	114	0	9211	96.53%	269	24	0	3980	94.97%
100	Overall accuracy = 90.94%, kappa coefficient = 0.854						Overall accuracy = 92.54%, kappa coefficient = 0.884				
	Unclassified	0	0	0	0		0	0	0	0	
	Fields	15,567	382	436	787	91.03%	6748	106	49	416	91.47%
	Forest	457	31,397	1457	25	95.02%	209	13,518	318	17	95.56%
	Shrubs	856	1200	3612	19	65.61%	361	518	1691	14	82.17%
	Urban	221	65	0	8711	91.29%	59	4	0	3744	89.33%
200	Overall accuracy = 91.37%, kappa coefficient = 0.865						Overall accuracy = 93.77%, kappa coefficient = 0.902				
	Unclassified	0	0	0	0		0	0	0	0	
	Fields	16,385	778	1166	516	95.81%	7102	274	296	134	96.27%
	Forest	290	31,580	1733	25	95.57%	116	13,565	24	414	95.89%
	Shrubs	151	605	2606	5	47.34%	84	12	3866	0	92.25%
	Urban	275	81	0	8996	94.28%	75	295	5	1510	73.37%
300	Overall accuracy = 91.91%, kappa coefficient = 0.875						Overall accuracy = 93.48%, kappa coefficient = 0.899				
	Unclassified	0	0	0	0		0	0	0	0	
	Fields	16,291	574	800	596	95.26%	7067	187	329	71	95.8%
	Forest	153	30,836	819	16	93.32%	86	13,244	15	170	93.62%
	Shrubs	418	1555	3886	24	70.59%	67	13	3832	0	91.43%
	Urban	239	80	0	8906	93.33%	157	702	15	1817	88.29%

**Figure 6:**
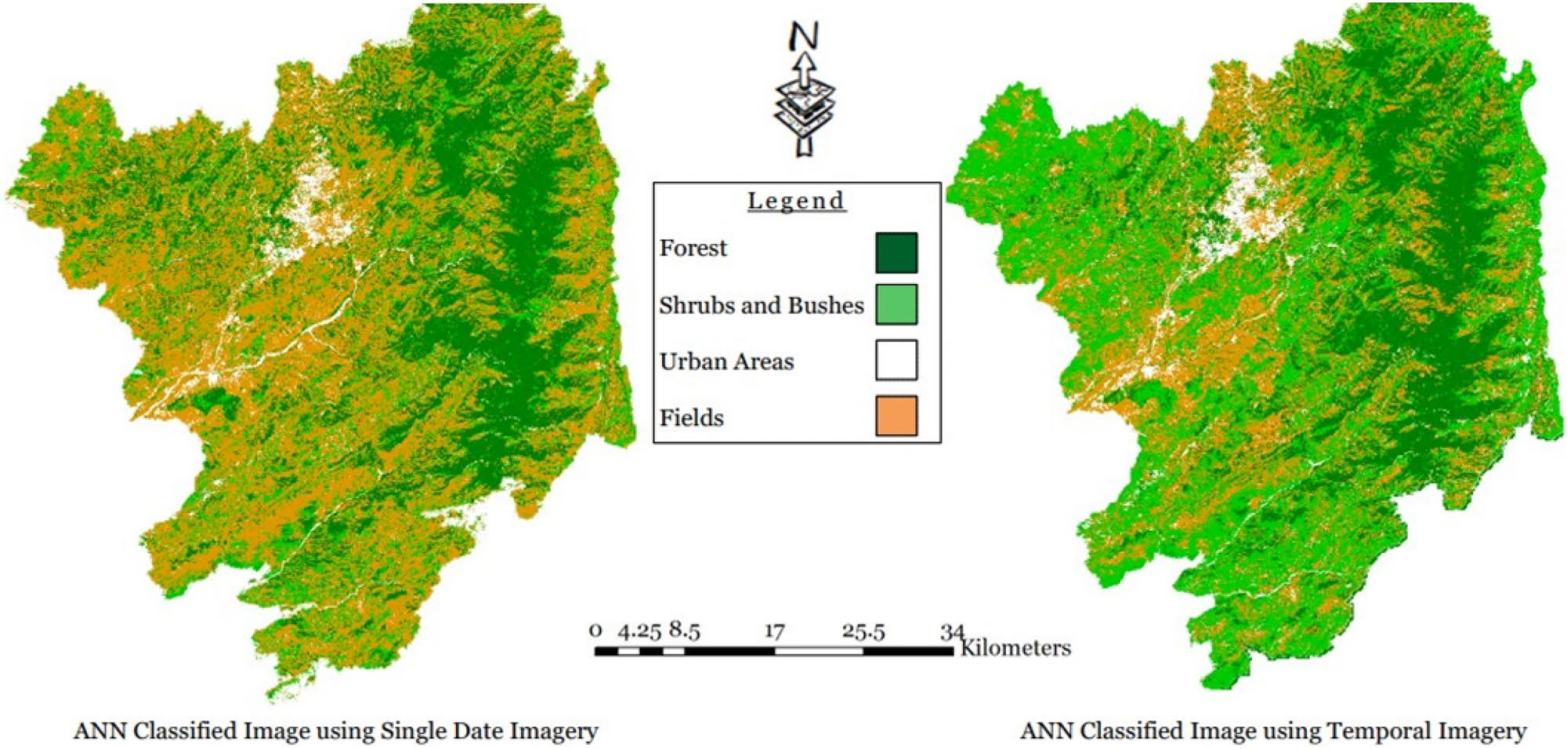
Neural network algorithm results for single and temporal layer-stacked image.

Figure 6 visually depicts the results of the ANN algorithm with 200 total iterations in the single-layer stacked images, where the yellow color represents fields areas, green indicates forest areas, light green corresponds to shrubs areas, and white denotes urban areas in District Abbottabad.

To improve result accuracy, a Temporal layer stack image of the District Abbottabad region was employed with Artificial Neural Network supervised classification algorithms, as outlined in Table 5. For a Temporal image, 70% of the data was designated for Training, while 30% was utilized for Testing or Validation to evaluate accuracy. With a total of 50 iterations for the Temporal layer stack image, the experimental outcomes demonstrated an Overall Training accuracy of 97.07% and a Testing or Validation accuracy of 97.29%. The Kappa Coefficient for 50 iterations was 0.954 in the Training data and 0.957 in the Testing data.

**Table 5:** ANN algorithm for temporal layer stacked image.

ANN results for temporal layer stacked image											
Training 70							Testing 30				
No of training		Fields_ training	Forest_ training	Shrubs_ training	Urban_ training	Overall. accuracy	Fields_ testing	Forest_ testing	Shrubs_ testing	Urban_ testing	Overall. accuracy
50	Overall accuracy = 97.07%, kappa coefficient = 0.954						Overall accuracy = 97.29%, kappa coefficient = 0.957				
	Unclassified	0	0	0	0		0	0	0	0	
	Fields	16,499	252	267	105	96.48%	15,922	791	1019	304	93.11%
	Forest	165	32,607	464	21	98.68%	207	31,004	1149	17	93.83%
	Shrubs	44	87	4759	2	86.45%	284	1135	3337	10	60.62%
	Urban	393	98	15	9414	98.66%	688	114	0	9211	96.53%
100	Overall accuracy = 97.73%, kappa coefficient = 0.964						Overall accuracy = 97.43%, kappa coefficient = 0.959				
	Unclassified	0	0	0	0		0	0	0	0	
	Fields	16,412	171	75	101	95.97%	7046	30	47	46	95.51%
	Forest	291	32,653	196	19	98.82%	131	13,992	129	7	98.91%
	Shrubs	114	121	5229	3	94.99%	33	99	1882	1	91.45%
	Urban	284	99	5	9419	98.71%	167	25	0	4137	98.71%
200	Overall accuracy = 98.07%, kappa coefficient = 0.970						Overall accuracy = 97.75%, kappa coefficient = 0.965				
	Unclassified	0	0	0	0		0	0	0	0	
	Fields	16,720	229	68	132	97.77%	7179	56	62	44	97.32%
	Forest	69	32,514	124	18	98.4%	43	13,898	5	66	98.25%
	Shrubs	120	210	5313	4	96.51%	113	20	4123	0	98.38%
	Urban	192	91	0	9388	98.39%	42	172	1	1948	94.66%
300	Overall accuracy = 97.69%, kappa coefficient = 0.964						Overall accuracy = 97.44%, kappa coefficient = 0.960				
	Unclassified	0	0	0	0		0	0	0	0	
	Fields	16,959	396	225	346	99.17%	7293	119	181	87	98.86%
	Forest	28	32,550	272	20	98.5%	29	13,985	7	188	98.86%
	Shrubs	21	51	5008	3	90.97%	40	9	4001	0	95.47%
	Urban	93	48	0	9173	96.13%	15	33	2	1783	86.64%

Upon increasing the total iterations to 100, a Training accuracy of 97.73% and a Testing Overall accuracy of 97.43% were attained. The Kappa Coefficient for 100 iterations was 0.964 in the Training data and 0.959 in the Testing data. Similarly, employing a total of 200 iterations for the layer stack image resulted in a Training accuracy of 98.07% and a Testing accuracy of 97.75%. The Training Kappa Coefficient was 0.970, and the Testing Kappa Coefficient was 0.965 for 200 iterations.

Finally, with a total of 300 iterations, an Overall Training accuracy of 97.69% and a Testing Overall accuracy of 97.44% were achieved. The Kappa Coefficient for 300 Training iterations was 0.964, and for Testing 300 iterations, it was 0.960. Figure 6 visually represents the results of the Temporal layer stacked image using the Neural Network algorithm.

In the exploration of the Random Forest Algorithm using Sentinel-2 single-layer stacked image data from the Abbottabad region, we observed notable variations in performance with different Maximum Depth parameters. Beginning with a setting of 5 Maximum Depth parameters for Training data, we achieved an Overall Training accuracy of 88.43% and a Testing/Validation accuracy of 91%. The Kappa Coefficient for Training data stood at 0.793, and for Testing data, it reached 0.846, as detailed in Table 6.

**Table 6:** Random forest algorithm results for single layer stacked image.

Random forest for single image											
Training 70							Testing 30				
Maximum depth		Fields_ training	Forest_ training	Shrubs_ training	Urban_ training	Overall. accuracy	Fields_ testing	Forest_ testing	Shrubs_ testing	Urban_ testing	Overall. accuracy
5	Overall accuracy = 88.43%, kappa coefficient = 0.793						Overall accuracy = 91.00%, kappa coefficient = 0.846				
	Unclassified	0	0	0	0		0	0	0	0	
	Fields	14,892	1317	488	403	84.22%	6473	515	220	168	88.14%
	Forest	637	31,320	1035	52	91.89%	194	13,469	475	7	94.7%
	Shrubs	1328	1449	2727	0	64.06%	283	239	1535	0	68.65%
	Urban	826	0	7	8708	95.03%	394	0	6	3790	95.59%
10	Overall accuracy = 92.83%, kappa coefficient = 0.881						Overall accuracy = 92.58%, kappa coefficient = 0.877				
	Unclassified	0	0	0	0		0	0	0	0	
	Fields	15,922	539	434	205	90.20%	6654	320	239	163	91.35%
	Forest	482	31,896	604	62	95.14%	171	13,590	373	11	95.51%
	Shrubs	752	1089	3663	0	77.80%	174	306	1577	0	71.84%
	Urban	496	2	7	9036	97.13%	285	12	6	3887	95.71%
20	Overall accuracy = 99.12%, kappa coefficient = 0.986						Overall accuracy = 92.90%, kappa coefficient = 0.882				
	Unclassified	0	0	0	0		0	0	0	0	
	Fields	17,027	20	50	3	98.09%	6706	273	226	171	92.55%
	Forest	67	32,934	43	0	99.57%	157	13,664	310	14	95.13%
	Shrubs	122	123	5259	0	98.26%	172	411	1474	0	73.00%
	Urban	143	0	0	9398	99.97%	211	15	9	3955	95.53%

**Figure 7:**
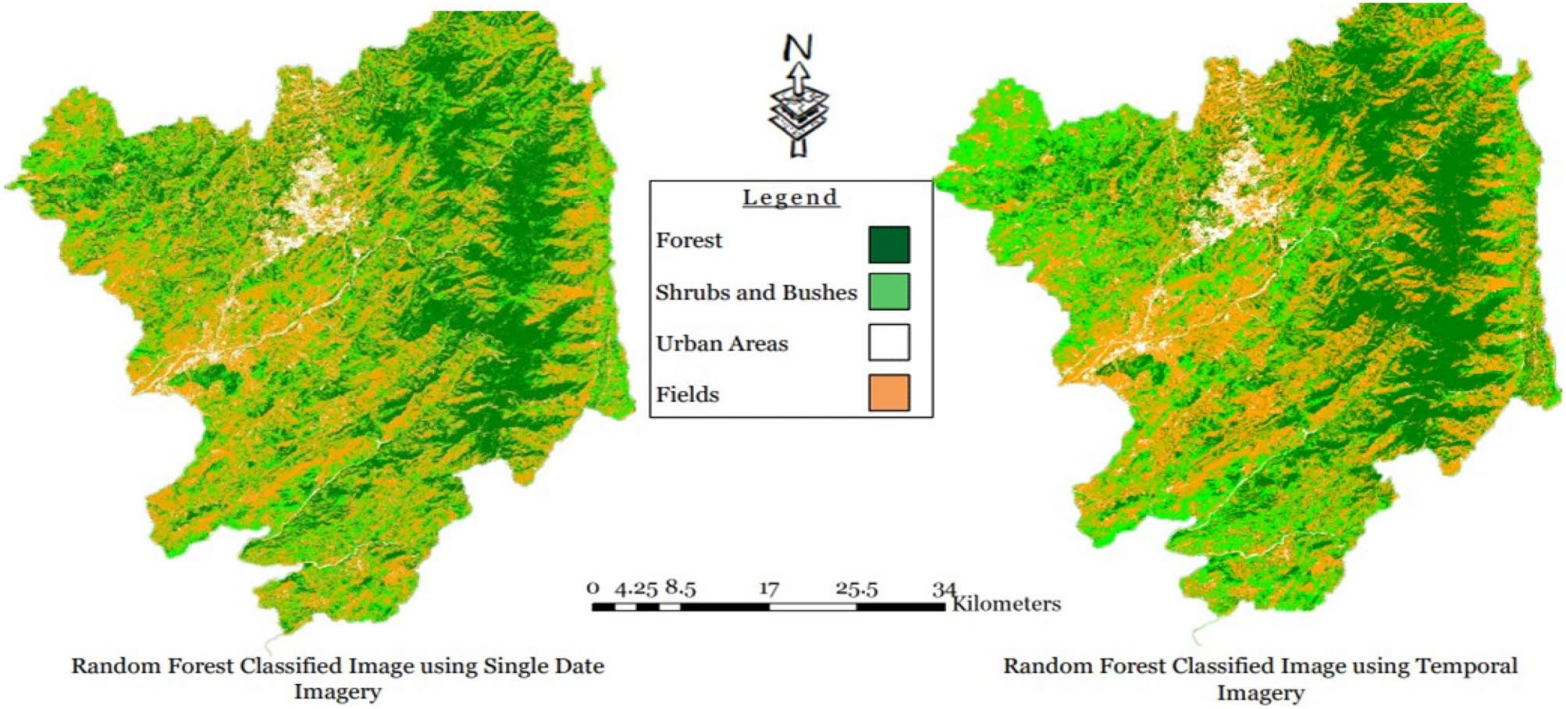
Random forest algorithm results for single and temporal layer-stacked image.

Advancing to a higher setting of 10 Maximum Depth parameters, a significant improvement was observed. The Overall Training accuracy surged to 92.83%, and the Testing/Validation accuracy reached 92.58%. The Kappa Coefficient for Training data rose to 0.881, while for Testing data, it increased to 0.877. Further increasing the Maximum Depth parameters to 20 resulted in remarkable outcomes. The Overall Training accuracy soared to 99.12%, and the Testing Overall accuracy remained high at 92.90%. The Kappa Coefficient for Training data achieved an impressive 0.986, and for Testing data, it maintained a substantial level at 0.882. These results collectively indicate that employing 20 Maximum Depth parameters yields the most favorable outcome for the Sentinel-2 single-layer stacked image data in the Abbottabad region, as illustrated in Fig. 7.

Concluding the experiments, the Random Forest algorithm was applied to the Temporal Layer stacked image of the Sentinel-2 image in the Abbottabad region, with a split of 70% for Training data and 30% for Testing data. The results, depicted in Table 7, showcased state-of-the-art performance. Initially, setting the Maximum Depth to 5 yielded an impressive 94.07% Overall Training accuracy and 94.79% Overall Validation accuracy. The Kappa Coefficient reached 0.903 for Training data and 0.916 for Testing data. Subsequently, with the Maximum Depth set to 10, the model achieved even higher accuracy, with 97.49% for Training and 96.33% for Testing or Validation. The Kappa Coefficient improved to 0.961 for Training and 0.942 for Testing data. Finally, pushing the Maximum Depth to 20 resulted in exceptional accuracy, with 99.79% for Training and 96.98% for Testing or Validation accuracy, as illustrated in Fig. 7. The Kappa Coefficient demonstrated remarkable values, standing at 0.996 for Training data and 0.954 for esting data, as detailed in Table 7.

**Table 7:** Random forest algorithm results for temporal layer stacked image.

Random forest algorithm results for temporal layer stacked image											
Training 70							Testing 30				
Maximum depth		Fields_ training	Forest_ training	Shrubs_ training	Urban_ training	Overall. accuracy	Fields_ testing	Forest_ testing	Shrubs_ testing	Urban_ testing	Overall. accuracy
5	Overall accuracy = 94.07%, kappa coefficient = 0.903						Overall accuracy = 94.79%, kappa coefficient = 0.916				
	Unclassified	0	0	0	0		0	0	0	0	
	Fields	16,540	182	144	234	89.98%	7079	98	92	107	91.06%
	Forest	622	32,050	326	46	96.22%	210	13,812	116	7	97.04%
	Shrubs	761	1076	3667	0	88.40%	276	323	1458	0	87.15%
	Urban	459	0	11	9071	97.01%	209	0	7	3974	97.21%
10	Overall accuracy = 97.49%, kappa coefficient = 0.961						Overall accuracy = 96.33%, kappa coefficient = 0.942				
	Unclassified	0	0	0	0		0	0	0	0	
	Fields	16,849	58	65	128	95.53%	7140	81	59	96	94.37%
	Forest	298	32,536	159	51	98.7%	121	13,901	111	12	97.83%
	Shrubs	260	377	4867	0	95.68%	171	221	1665	0	90.49%
	Urban	231	1	5	9304	98.11%	134	7	5	4044	97.40%
20	Overall accuracy = 99.79%, kappa coefficient = 0.996						Overall accuracy = 96.98%, kappa coefficient = 0.954				
	Unclassified	0	0	0	0		0	0	0	0	
	Fields	17,063	0	6	31	99.56%	7158	91	33	94	96.18%
	Forest	36	32,986	15	7	100%	69	13,977	60	12	97.74%
	Shrubs	6	0	5498	0	99.62%	114	227	1716	0	94.70%
	Urban	33	0	0	9508	99.60%	101	5	3	4081	97.47%

**Figure 8:**
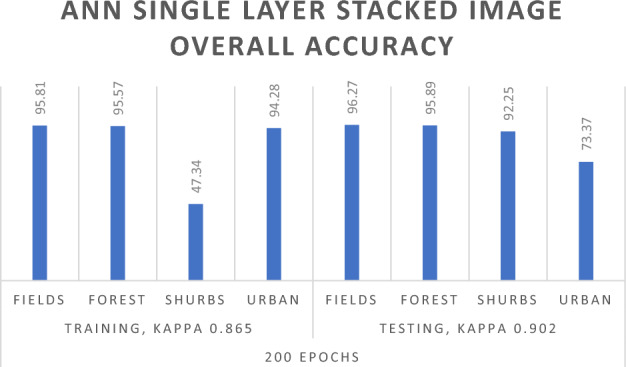
ANN single layer stacked overall accuracy with 200 iteration.

**Figure 9:**
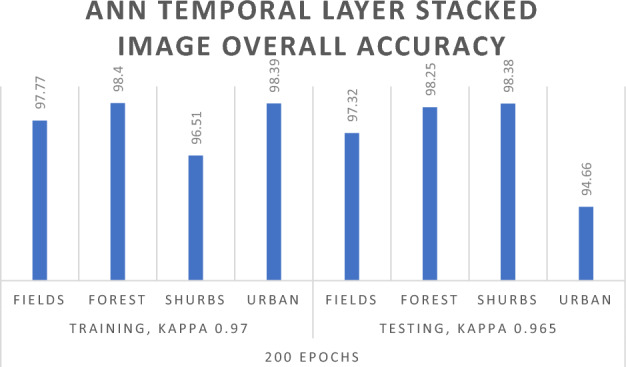
AAN temporal layer stacked overall accuracy with 200 iteration.

The experimental results of the neural network for both single-layer stacked images and temporal images in Table 4 and Table 5 reveal a positive correlation between the total number of iterations and algorithm performance. An enhancement in performance is observed with an increase in the total number of iterations, reaching the best result at 200 total iterations for both single and Temporal images In Figs. 8 and 9. Nevertheless, an important observation is that surpassing 200 total iterations results in a decline in the performance of the neural network algorithm. This decrease is clearly reflected in both Tables 4 and 5, where, with 300 total iterations, the Validation Overall accuracy shows a reduction. One plausible explanation for this trend could be that networks with a substantial number of parameters are susceptible to overfitting in Neural Network. Overfitting occurs when a model captures noise or specific patterns in the Training data that do not generalize effectively to new, unseen data. To solve the overfitting problem, we used the Random Forest algorithm.

**Figure 10:**
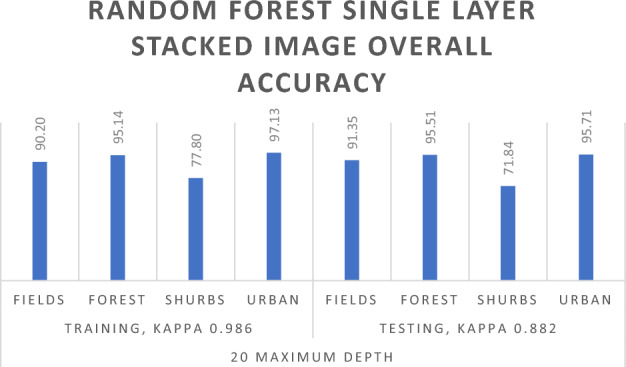
RF single layer stacked overall accuracy with 20 maximum depth.

**Figure 11:**
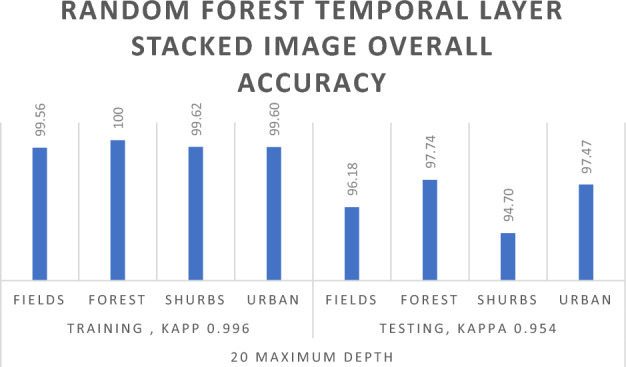
RF temporal layer stacked overall accuracy with 20 maximum depth.

Based on the outcomes of the Random Forest algorithm applied to both single-layer and temporal layer stacked images, it is deduced that enhancing the Maximum Depth contributes to improved results as shown in Table 6. Specifically, the optimal outcome for the single-layer stacked image is achieved with a Maximum Depth of 20, as illustrated in Fig. 10. Similarly, for the temporal layer stacked image, superior results are attained by increasing the Maximum Depth. The most favorable outcome for the Random Forest algorithm in the temporal layer stacked image is observed with a Maximum Depth of 20, as indicated in Table 7 and in Fig. 11.

Applying the Neural Network algorithm with 200 total iterations to a 166,103-hectare area in the Abbottabad region, as detailed in Table 8 for the Temporal Layer Stacked Image, reveals a forested area spanning 51,613 hectares. The percentage representation of forest cover in the Abbottabad region is determined to be 31.07%. In the assessment of the total forest area using the Random Forest algorithm with a Maximum Depth of 20, the analysis was conducted on a 166,120-hectare area, as specified in Table 9. Within this area, the forested region is measured at 51,774 hectares. The percentage representation of forest cover in the Abbottabad region is calculated to be 31.17%.

**Table 8:** Total area of Abbottabad by using neural network algorithm.

Class	Area (ha)	Percentage (%)
Fields	62,151	37.42
Forest	51,613	31.07
Shrubs	44,335	26.70
Urban	8004	4.82
Total area	166,103

**Table 9:** Total area of Abbottabad by using random forest algorithm.

Class	Area (ha)	Percentage (%)
Fields	68,379	41.16
Forest	51,774	31.17
Shrubs	39,218	223.61
Urban	6749	4.06
Total area	166,120	

## Discussion

In contrast to previous studies highlighted in the literature review as illustrated in Table 8, our work has demonstrated notably superior results due to the accumulation of structurally enriched satellite images. The adoption of relevant Training images has improved the existing results previously attained in the baseline methods as is shown in Table 10. Notably, when employing the Random Forest algorithm and Sentinel-2 data, Li et al.^5^ achieved an accuracy of 95.76%, and Kappa Coefficient 0.91 whereas our approach yielded a higher Overall accuracy of 96.98% and 0.95 Kappa Coefficient as depicted in Tables 3 and 5. Additionally, in comparison to Moradi et al.^30^, who attained a 97% Overall accuracy and 0.94 Kappa Coefficient using Landsat data and CNN, our methodology utilizing an Artificial Neural Network (ANN) algorithm and Sentinel-2 data surpassed this performance with an Overall accuracy of 97.75% and 0.965 Kappa Coefficient.

**Table 10:** Results from existing literature review.

Authors	Satellite/data/algorithms	Results
Li et al.^5^	Sentinel 2, Forest, Random Forest	95.76% overall accuracy Kappa coefficient 0.91
Liu et al.^6^	Landsat, LULC, Random Forest	91.41% overall accuracy Kappa coefficient 0.86
Erfanifard et al.^15^	Landsat, Mangrove Forest, SMRI	97% overall accuracy Kappa coefficient 0.96
Islam et al*.*^17^	Landsat, Mangrove Forest, SAVI and NDVI	87% overall accuracy Kappa coefficient 0.84
Negassa et al.^20^	Landsat, Forest, Random Sampling Technique	85% overall accuracy Kappa coefficient 0.86
Yuh et al.^29^	Landsat 7 ETM and Landsat 8 OLI, LULC, RF, ANN, KNN, SVM	95.8% overall accuracy (ANN) Kappa coefficient 0.94
Moradi et al.^30^	Landsat TM, Landsat ETM, Forest, CNN	97% overall accuracy (ANN) Kappa coefficient 0.94

## Conclusion

Pakistan faces challenges as a forest-poor country, with less than 6% of its total area covered by forests. To address this issue, advanced techniques employing Machine Learning and Deep Learning algorithms were applied for forest cover classification in District Abbottabad. Notably, the Artificial Neural Network (ANN) and Random Forest algorithms were employed, yielding state-of-the-art results in terms of Overall Accuracy and Kappa Coefficient. The ANN algorithm demonstrated remarkable performance, achieving a best Overall Accuracy of 97.75% and a Kappa Coefficient of 0.965. However, to tackle the overfitting problem inherent in ANN, the Random Forest algorithm was introduced. This approach resulted in a commendable Overall Accuracy of 96.98% and a Kappa Coefficient of 0.954, particularly when using a maximum depth of 20 for the Temporal Layer stacked image. Applying the ANN algorithm to the entire 166,103 hectares area of Abbottabad revealed a forest cover of 51,613 hectares, constituting 31.07% of the total district area. Meanwhile, utilizing the Random Forest algorithm identified a total forest cover of 51,774 hectares, equivalent to 31.17% of the Abbottabad district region. To elevate the precision of forest cover classification, incorporating hyperspectral satellite imagery is recommended. Additionally, for future enhancements, deep learning algorithms such as Convolutional Neural Networks (CNN), Long Short-Term Memory networks (LSTM), and Gated Recurrent Units (GRU) will be explored for their potential in advancing classification accuracy.

## Data Availability

The datasets used and/or analysed during the current study are available from the corresponding author on reasonable request.
